# Spectrum Handoff Based on DQN Predictive Decision for Hybrid Cognitive Radio Networks

**DOI:** 10.3390/s20041146

**Published:** 2020-02-19

**Authors:** Kaitian Cao, Ping Qian

**Affiliations:** 1 School of Electrical & Electronic Engineering, Shanghai Institute of Technology, Shanghai 201418, China; qping@sit.edu.cn; 2 Key Laboratory of Broadband Wireless Communication and Sensor Network Technology, Ministry of Education, Nanjing University of Posts and Telecommunications, Nanjing 210003, China

**Keywords:** cognitive radio networks, spectrum handoffs, machine learning, deep Q-network, transfer learning

## Abstract

Spectrum handoff is one of the key techniques in a cognitive radio system. In order to improve the agility and the reliability of spectrum handoffs as well as the system throughput in hybrid cognitive radio networks (HCRNs) combing interweave mode with underlay mode, a predictive (or proactive) spectrum handoff scheme based on a deep Q-network (DQN) for HCRNs is proposed in this paper. In the proposed spectrum handoff approach, spectrum handoff success rate is introduced into an optimal spectrum resource allocation model to ensure the reliability of spectrum handoff, and the closed-form expression for the spectrum handoff success rate is obtained based on the Poisson distribution. Furthermore, we exploit the transfer learning strategy to further improve the DQN learning process and finally achieve a priority sequence of target available channels for spectrum handoffs, which can maximize the overall HCRNs throughput while satisfying constraints on secondary users’ interference with primary user, limits on the spectrum handoff success rate, and the secondary users’ performance requirements. Simulation results show that the proposed spectrum handoff scheme outperforms the state-of-the-art spectrum handoff algorithms based on predictive decision in terms of the convergence rate, the handoff success rate and the system throughput.

## 1. Introduction

Cognitive radio networks (CRNs) have received great attention due to their potential to provide an efficient solution to the contradiction between spectrum scarcity and inefficient spectrum utilization, and improve system capacity via dynamic spectrum access (DSA) and spectrum management techniques [1,2]. Therefore, efficient spectrum management and resource allocation are crucial for CRNs to solve the shortage of spectrum resources and improve spectrum utilization [3,4]. DSA in CRNs can be categorized into three modes: overlay, interweave, and underlay [4,5,6]. Theoretically, underlay mode can significantly improve spectrum efficiency due to the fact that it allows secondary users (SUs) to access the licensed spectrum along with active primary users (PUs) at the same time [4,5,6,7,8]. In interweave mode [5,6,9], SUs can be allowed to access the vacant spectrum which is not occupied by any active PUs. Whenever a PU becomes active, SUs must vacate the licensed spectrum immediately. In overlay mode, SUs are allowed to simultaneously share the licensed spectrum bands with PUs without imposing any constraint on SUs’ transmission power if SUs have a full knowledge of PUs’ signals characteristics [5,6], which is infeasible in practice due to the difficulty to obtain all the prior knowledge of PU’s signals. Compared to overlay mode, interweave and underlay technologies can achieve higher spectrum utilization. Currently, there is hardly any consensus on which of the two spectrum access modes (interweave and underlay) is more suitable for CRNs system [9]. Therefore, it is of more theoretical and practical significance to investigate hybrid CRN (HCRN) technology which is a mixed spectrum sharing mode by combining the interweave mode and underlay mode.

However, in order to achieve efficient spectrum utilization, HCRN systems face many technical challenges, one of which is spectrum handoff technology [10,11]. In HCRNs, when the channel performance deteriorates, or the SUs’ interference with PU exceeds the PU’s tolerance threshold, SUs have to vacate and switch to a new target channel to continue data transmission. According to the decision timing for selecting target channels, spectrum handoff methods can be classified into the reactive decision based and the proactive (or predictive) decision-based handoffs [11,12,13]. Predictive decision can select a series of prospective backup vacant target channels before spectrum handoff occurs, which can save substantial sensing time. Therefore, the predictive decision-based spectrum handoff schemes have become a research focus of CRNs [11,13]. In [14], Hoque et al. established an analytical mode for the probability of spectrum handoff and further derived an analytical expression for average spectrum handoff number for a SU based on the residual time distributions of spectrum holes, and investigated the effect of spectrum handoff delay on the performance of spectrum mobility in CRNs. However, [14] does not take into account the overall optimization problems, such as the SU’s transmission rate, system throughput, and so on. The work in [15] developed an analytical model for the general case of non-identical channels in CRNs, and introduced the model for both fixed and probabilistic sequence approaches for target channel selection. In [16], authors proposed an adaptive hybrid spectrum sharing method based on the rate compensation approach and adapted best fit algorithms, taking static and dynamic spectrum sharing algorithms into consideration. In [17], Kumar et al. presented a proactive decision-based spectrum handoff algorithm by utilizing multi-attribute decision making method according to different requirements of network service. In [11], a proactive decision based-handoff scheme (PDBHS) for CRNs is proposed, in which a hybrid handoff strategy is addressed by minimizing the number of handoffs such that the total service time is minimized. PDBHS requires *K* fixed slots for spectrum handoff, and the available target idle channels are sorted in a decreasing order of probability of obtaining *K* consecutive idle time slots. However, since the spectrum handoff time of PDBHS is fixed as *K* time slots, it is impossible for PDBHS to really reduce the service time. Besides, some performance challenges such as the maximum system capacity are not considered in the PDBHS scheme.

In addition, the above spectrum handoff methods based on predictive decision still have the following drawbacks: (1) data transmission only between a pair of sending and receiving SUs is considered, however, the impact of surrounding SUs’ behaviors on a SU is not taken into account; (2) only the spectrum handoff scenario in a single spectrum access mode is considered for CRNs, but the hybrid spectrum access scenario combining interweave mode with underlay mode as well as the multi-SU spectrum handoff problem are not addressed; (3) the spectrum handoff success rate or failure rate is not investigated yet.

In order to solve the shortcomings of the above existing spectrum handoff approaches, we propose a transfer learning (TL)-based predictive decision spectrum handoff (TL-PDSH) method by introducing a deep Q-network (DQN) [18], TL [19] strategy, and the handoff success rate in this paper.

Our main contributions are briefly summarized as follows:

(1) Spectrum handoff success rate is introduced, and a multi-SU DQN learning-based spectrum handoff method for HCRNs is developed.
(2) This paper develops an overall throughput optimization model for spectrum handoffs in HCRNs while meeting constraints on signal-to-interference plus noise ratio (SINR) thresholds, the level of SUs’ interference with PU, and requirements for the handoff success rate.
(3) A DQN algorithm is used to obtain the optimal learning strategy and seek the target channel sequence for spectrum handoffs, and the TL strategy is introduced in our method to further improve the DQN learning process.

## 2. System Model

In this paper, we study HCRNs where two central wireless networks, a primary network and a secondary network, share the licensed spectrum in a hybrid way combing interweave with underlay. In this scenario, SUs and PUs are randomly located around secondary base station (SBS) and primary base station (PBS), respectively.

In this paper, we assume that a SU senses a set of *N*+1 non-overlapping PU channels among which one PU channel is being occupied by the SU and *N* is the number of remaining PU channels that are arranged as ${\left\{\phi_{i}\right\}}_{i=1}^{N}$ in an increasing order of their central frequencies. Before spectrum handoff occurs, it is assumed that SBS obtains the state information of PU channels in advance. In our spectrum handoff scheme, SBS predicts the *N* channels capacity and selects *M* available target channels ${\left\{\varphi_{i}\right\}}_{i=1}^{M}$ (*M ≤ N*) ready for spectrum handoff from the *N* channels in which the interference caused to PU is below a certain threshold. Therefore, the proposed TL-PDSH method in this paper can predict the channel capacity of ${\left\{\varphi_{i}\right\}}_{i=1}^{M}$ and obtain the handoff priority sequence ${\left\{\omega_{i}\right\}}_{i=1}^{M}$ which is rearranged in a decreasing order of the channel capacity of ${\left\{\varphi_{i}\right\}}_{i=1}^{M}$.

We assume that the process of PU appearing in a licensed channel is a Poisson process with appearance rate 1/λ, then the time interval the channel remaining idle, denoted by X, obeys a Poisson distribution, and its probability density function (PDF) is:

(1) $$f(x)=\left\{ \begin{array}{l} \frac{1}{\lambda }e^{-x/\lambda },x>0\\ 0,\;\mathrm{others} \end{array}\right.$$

where λ=E(X), E(⋅) is the expectation operator. For brevity, the idle time durations of the target channel sequence ${\left\{\omega_{i}\right\}}_{i=1}^{M}$ are denoted as ${\left\{\lambda_{i}\right\}}_{i=1}^{M}$, respectively. 

In HCRNs, when the licensed channel is not occupied by a PU, SUs can achieve the maximum transmission rate over the channel unoccupied by PU since the interference caused by the PU to SUs is minimal. In other words, the higher the probability that a PU channel is vacant is, the higher the probability of SU’s switching to the vacant channel is. Therefore, in order to obtain the optimal overall throughput of HCRNs system, all SUs prefer to choose the licensed channels unoccupied by PUs as the target channels for the upcoming spectrum handoffs. Motivated by this, this paper introduces a spectrum handoff success rate denoted as $P_{s}$ to characterize the above phenomena. From the Poisson distribution, it is easy to deduce the handoff success rate (the overall idle probability of the channel) on the target channel sequence ${\left\{\omega_{i}\right\}}_{i=1}^{M}$ as follows:

(2) $$P_{s}=1-\prod_{i=1}^{M}\left(1-e^{-\left[(i-1)T_{H}+T_{ACK}\right]/\lambda_{i}}\right)$$

where *T_H_* is the period of handoffs, *T_ACK_* denotes the time duration of spectrum handoff acknowledge.

In this paper, we assume that PUs and SUs transmit using adaptive modulation and coding (AMC) [20] technology. Through AMC technique, SBS can infer the channel state information (CSI) of both PU and SU by active learning, estimate some parameters such as the channel gains [21], and dynamically adapt their own parameters to meet the constraints on the interference with primary link. Therefore, In HCRNs, in order to avoid the impact of each SU’s behavior on primary link, the total interference caused by all SUs to PU must be limited below an allowable level. 

In AMC, the modulation methods and channel coding rates can be adjusted adaptively according to SINRs. By using AMC technique, the SINR measured for the PU at PBS, denoted as $SINR^{\left(p\right)}$, and for the SU*_i_* (*i*=1, 2,…, *L*) at SBS, denoted as $SINR_{i}^{\left(s\right)}$, which can be expressed as follows:

(3) $$SINR^{\left(p\right)}=\frac{G_{0}^{\left(p\right)}P_{0}}{\sigma^{2}+\sum_{i=1}^{L}G_{i}^{\left(p\right)}P_{i}}$$

(4) $$SINR_{i}^{\left(s\right)}=\frac{G_{i}^{\left(s\right)}P_{i}}{\sigma^{2}+G_{0}^{\left(s\right)}P_{0}+\sum_{j\neq i}^{L}G_{j}^{\left(s\right)}P_{j}}$$

where *L* is the number of SUs, $G_{0}^{\left(p\right)}$ and $G_{i}^{\left(p\right)}$ are the channel gain between the PU and PBS, and SU*_i_* ’s channel gain to PBS, respectively. $G_{i}^{\left(s\right)}$ and $G_{j}^{\left(s\right)}$ denote SU*_i_*’s channel gain to SBS, and SU*_j_* (*j* ≠ *i*) to SBS channel gain, respectively. $G_{0}^{\left(s\right)}$ is the channel gain between the PU and SBS. $P_{0}$, $P_{i}$, and $P_{j}$ are the transmitted powers by PU, SU*_i_*, and SU*_j_*, respectively. $\sigma^{2}$ is the AWGN power.

In HCRNs, in order to ensure the communication performance of PU and SUs, constraints should be imposed on the SINRs for both PU and SU*_i_*, which can be formalized by introducing SINR thresholds $\mu_{0}$ and $\mu_{i}$ as:

(5) $$SINR^{\left(p\right)}\geq \mu_{0}$$

(6) $$SINR_{i}^{\left(s\right)}\geq \mu_{i},i=1,2,\ldots ,L$$

According to the power allocation scheme in [22], we can obtain the power $P_{i}$ allocated to SU*_i_* as below:

(7) $$P_{i}=\frac{\beta_{i}\left(\sigma^{2}+G_{0}^{\left(s\right)}P_{0}\right)}{G_{i}^{\left(s\right)}\left(1-\sum_{i=1}^{L}\beta_{i}\right)},i=1,2,\ldots ,L$$

where:

(8) $$\beta_{i}={\left(1+1/\mu_{i}\right)}^{-1}$$

and:

(9) $$1-\sum_{i=1}^{L}\beta_{i}>0$$

Substituting Equation (7) into Equations (3) and (4), then Equations (5) and (6) can be uniformly expressed as:

(10) $$\sum_{i=1}^{L}\alpha_{i}\beta_{i}\leq 1$$

where:

(11) $$\alpha_{i}=\frac{G_{i}^{\left(p\right)}\left(\sigma^{2}+G_{0}^{\left(s\right)}P_{0}\right)}{G_{i}^{\left(s\right)}\left(-\sigma^{2}+G_{0}^{\left(p\right)}P_{0}/\mu_{0}\right)}+1$$

From Equations (7) and (10), the SINR threshold $\mu_{i}$ needs to be adjusted dynamically in order to satisfy the constraint on the secondary link’s interference with PU and maximize the overall throughput of HCRNs. In HCRNs, the SU*_i_*’s transmit bit rate $R_{i}$ [20] can be expressed as:

(12) $$R_{i}=W\log_{2}(1+k\mu_{i})$$

where W is the channel bandwidth, $k=-1.5/\ln(5r_{b})$ is a constant determined by the maximum transmit bit error rate $r_{b}$.

## 3. TL-PDSH Spectrum Handoff Scheme Based on DQN

When SUs’ interfere with PU, the success rate of the spectrum handoff and other conditions are satisfied, and in order to maximize the overall throughput of HCRNs system, the SINR threshold $\mu_{i}$ needs to be adjusted dynamically. Therefore, how to choose $\mu_{i}$ becomes crucial. The selection problem of the optimal SINR threshold $\mu_{i}^{*}$ can be formulated as:

(13) $$\begin{array}{l} \left\{\mu_{i}^{*}\right\}=\arg\max\frac{1}{L}\sum_{i=1}^{L}R_{i}\\ \begin{array}{ll} s.t. & \sum_{i=1}^{L}\alpha_{i}\beta_{i}\leq 1\\ & 1-\sum_{i=1}^{L}\beta_{i}>0\\ & P_{s}\geq \rho \end{array} \end{array}$$

where ρ is the minimum success rate of spectrum handoff. The constrained optimization expression in Equation (13) is actually an optimal resource allocation problem, which can maximize the overall throughput of the HCRN system while meeting the constraints on both the SINR thresholds and the successful handoff rate. Reinforcement learning (RL) has been proved to be an effective solution for the resource allocation problem in communication systems [23]. In this paper, we assume that the set of actions and the set of states in RL model are $A=\left\{a_{1},a_{2},\cdots a_{n}\right\}$ and $S=\left\{s_{1},s_{2},\cdots s_{m}\right\}$, respectively. At instant *t*, the RL agent takes an action a(t)∈A in the state s(t)∈S, receiving immediate reward r(s,a), and then the state is transited into the next state s(t+1)∈S. Q-learning is a classical RL algorithm, which first evaluates each action value (Q-value) of the learning agent and then obtains the optimal learning strategy based on Q-values.

However, Q-learning has two fatal disadvantages: (1) the sets of the states and actions applicable to Q-learning are very small; and (2) the predictive ability of Q-learning is very weak. To this end, in this paper, the TL-PDSH scheme establishes the action space, state space and reward function by introducing a neural network into the Q-learning method, yielding a DQN, and then a DQN algorithm is used to obtain the approximate estimator of Q-value and the optimal learning strategy. In order to maximize the overall throughput of HCRNs while meeting the constraints on SUs’ interference with Primary link, SU*_i_* (*i* = 1, 2,…, *L*) needs to seek a suitable SINR threshold $\mu_{i}$ within a certain range, and the set of these thresholds constitutes the action space $A_{i}$, denoted as $A_{i}=\left\{\mu_{i}^{\left(1\right)},\mu_{i}^{\left(2\right)},\cdots \right\}$ where $\mu_{i}^{\left(t\right)}$ is the SINR threshold at instant *t*. States can be defined as the three constraints in Equation (13), and then the state space at instant *t* can be formalized as $s^{\left(t\right)}=\left(I^{\left(t\right)},D^{\left(t\right)},G^{\left(t\right)}\right)$ where:

(14) $$I^{\left(t\right)}=\left\{ \begin{array}{l} 0,\;\sum_{i=1}^{L}\alpha_{i}\beta_{i}^{\left(t\right)}\leq 1\\ 1,\;\mathrm{others} \end{array}\right.$$

(15) $$D^{\left(t\right)}=\left\{ \begin{array}{l} 0,\;1-\sum_{i=1}^{L}\beta_{i}^{\left(t\right)}>0\\ 1,\;\mathrm{others} \end{array}\right.$$

(16) $$G^{\left(t\right)}=\left\{ \begin{array}{l} 0,\;P_{s}\geq \rho \\ 1,\;\mathrm{others} \end{array}\right.$$

The reward function is defined as a function in terms of the state space and the current action space, and then at instant *t*, SU*_i_* (*i*=1, 2,…, *L*) obtains the reward $r_{i}\left(s^{\left(t\right)},a^{\left(t\right)}\right)$ as follows:

(17) $$r_{i}\left(s^{\left(t\right)},a^{\left(t\right)}\right)=\left\{ \begin{array}{l} \Lambda ,\;I^{\left(t+1\right)}+D^{\left(t+1\right)}+G^{\left(t+1\right)}\geq 1\\ R_{i},\;\mathrm{others} \end{array}\right.$$

where Λ is a constant which is smaller than the reward received by an agent by taking any learning strategy. Therefore, Λ indicates that when any of the constraints in Equation (13) is not met, $r_{i}\left(s^{\left(t\right)},a^{\left(t\right)}\right)$ is a penalty, not a benefit.

It can be seen from the above analysis that since the transmit rate $R_{i}$ is positive, maximizing the overall throughput of HCRNs system is essentially to maximize the individual transmit rate $R_{i}$ of SU*_i_*. Therefore, the task of SU*_i_* is to seek an optimal learning policy π through DQN learning algorithm so as to maximize its reward at the next moment, namely:

(18) $$Q_{i}^{*}(s,a)=\underset{\pi }{\max}\{\sum_{t=0}^{\infty }[\gamma^{\left(t\right)}\times E\left(r_{i}\left(s^{\left(t\right)},a^{\left(t\right)}\right)|s^{\left(t\right)}=s,a^{\left(t\right)}=a,\pi \right)]\}$$

where $\gamma^{\left(t\right)}$ is the discounting factor at each time step *t*, E(⋅) is the expectation operator, $Q_{i}^{*}(s,a)$ is the optimal Q-value function of SU*_i_*, indicating the maximum sum of discounted rewards $r_{i}\left(s^{\left(t\right)},a^{\left(t\right)}\right)$ as t→∞, achieved by a behavior policy π. According to Bellman’s principle of optimality [24], if the optimal value $Q_{i}^{*}(s^{\prime },a^{\prime })$ of the state sequence $s^{\prime }$ at the next time step is known for all possible actions $a^{\prime }$, then Equation (18) can be rewritten as:

(19) $$Q_{i}^{*}(s,a)=E\left[r_{i}\left(s,a\right)+\gamma \;\underset{a^{\prime }}{\max}\;Q_{i}^{*}(s^{\prime },a^{\prime })\right]$$

From Equations (18) and (19), the iterative Equation (19) converges to the optimal Q-value only as t→∞, which is impractical since the Q-value function is estimated separately for each state in practice, without any generalization. Instead, in the TL-PDSH method presented in this paper, the DQN neural network has been utilized as an effective approximator to estimate the Q-value function $Q_{i}(s,a\begin{array}{c} ; \end{array}\theta_{i})\approx Q_{i}^{*}(s,a)$. In addition, the TL-PDSH method adopts a technique known as “experience replay” to improve learning performance. Different from the linear estimators widely used in the general RL methods, the TL-PDSH method is a nonlinear weighted approximator of the DQN neural network.

In experience replay, at each time step, SU*_i_* stores the experience values $e_{i}^{\left(t\right)}=\left[a_{i}^{\left(t\right)},s_{i}^{\left(t\right)},r_{i}^{\left(t\right)},s_{i}^{\left(t+1\right)}\right]$ interacting with the wireless environment into replay memory $M_{i}^{\left(t\right)}=\left\{e_{i}^{\left(0\right)},e_{i}^{\left(1\right)},\ldots ,e_{i}^{\left(t\right)}\right\}$ (*i*=1, 2,…, *L*). Suppose that $\theta_{i}^{-}$, $\theta_{i}$ are the previous parameter of Q-value and the updated parameter of Q-value, respectively, then $\theta_{i}$ can be updated by minimizing the following loss function $L(\theta_{i})$ under the current iteration step:

(20) $$L\left(\theta_{i}\right)=E\left[{\left(y_{i}-Q(s,a;\theta_{i})\right)}^{2}\right]$$

where $y_{i}=r_{i}\left(s,a\right)+\gamma \;\underset{a^{\prime }}{\max}\;Q_{i}(s^{\prime },a^{\prime };\theta_{i}^{-})$. In the proposed DQN-based TL-PDSH method, ϵ-greedy strategy [24] is used to select SU*_i_*’s action (SINR threshold $\mu_{i}$), updating the parameters $\theta_{i}$ so as to achieve the most reward for SU*_i_* and maximize the overall throughput of HCRNs system. Algorithm 1 displays main steps of our TL-PDSH scheme. Note that algorithm 1 does not introduce the TL strategy since it does not take into account the new SUs.
**Algorithm 1.** The proposed TL-PDSH scheme without TL (when no new SUs appear).**for** all SU*_i_*, *i* = 1,…, *L*
**do**   Initialize replay memory $M_{i}^{\left(0\right)}=\left\{e_{i}^{(0}\right\}$ at *t* = 0;   Initialize $\theta_{i}$ and γ;   Initialize the neural network for $Q_{i}(s,a\begin{array}{c} ; \end{array}\theta_{i})$ with $\theta_{i}$;   Initialize the neural network for $Q_{i}(s^{\prime },a^{\prime };\theta_{i}^{-})$ with $\theta_{i}^{-}$ = $\theta_{i}$;**end for****for***t* < T **do**   **for** all SU*_i_*, *i* = 1, …, *L*
**do**   Select a random action with probability ϵ (ϵ-greedy algorithm);   Otherwise select the action $a_{i}^{\left(t\right)}=\arg\max\;Q_{i}\left(s_{i}^{\left(t\right)},a_{i}^{\left(t\right)};\theta_{i}\right)$;   Update the state $s_{i}^{\left(t+1\right)}$ in (14)–(16) and the reward $r_{i}^{\left(t\right)}$;   Store $e_{i}^{\left(t\right)}=\left[a_{i}^{\left(t\right)},s_{i}^{\left(t\right)},r_{i}^{\left(t\right)},s_{i}^{\left(t+1\right)}\right]$ in $M_{i}^{\left(t\right)}$;   Update parameters of $Q_{i}(s,a\begin{array}{c} ; \end{array}\theta_{i})$ by minimizing $L\left(\theta_{i}\right)$ from $M_{i}^{\left(t\right)}$;   Update parameters of $Q_{i}(s^{\prime },a^{\prime };\theta_{i}^{-})$ with $\theta_{i}^{-}$=$\theta_{i}$ at each time step;   **end for****end for**

In addition, considering that the parameters of Q function of two adjacent SUs are similar in HCRNs system, therefore, TL algorithm [19] is exploited in our TL-PDSH scheme to initialize the parameters of the newcomer SU with the parameters of its nearest SU in HCRNs, instead of initiating the DQN learning process from scratch. As a result, TL-PDSH method can greatly speed up the DQN learning process and improve the performance of the whole HCRNs system by introducing TL strategy into the proposed spectrum handoff method. When a new SU joins the HCRN system, our proposed TL-PDSH scheme with TL is described in Algorithm 2.
**Algorithm 2.** The proposed TL-PDSH scheme with TL (when a new SU appears).A newcomer SU is denoted as SU*_L_*_+1_;Determine the nearest SU*s*(*i*) of SU*_L_*_+1_;Initialize *Q_L_*_+1_ with parameters of the *Q* value of the nearest neighbor *θ_L_*_+1_ =*θ_s_*_(*i*)_;Run **Algorithm 1**; /*(**Note:** now the number of SUs is (*L*+1))*/

## 4. Simulations Results

For convenience, the DQN-based TL-PDSH method proposed in this paper, the DQN-based PDSH algorithm without using the TL learning strategy, and the traditional PDSH algorithm based on Q-learning are denoted as TL-PDSH, PDSH, and Q-PDSH, respectively. In this section, the performance of TL-PDSH spectrum handoff method based on DQN is verified through Monte-Carlo simulations, and it is compared with the PDSH algorithm, the PDBHS algorithm [11], and the Q-PDSH method.

It is assumed that there is only one PU accessing a single channel in the primary network, the transmitting power of PBS is 100 mW, the Gaussian noise power is 10 nW, and the SINR for the PU is set at 1dB. The distance between PBS and SBS is 2 km, and PU and all SUs are randomly distributed around their respective base stations within a circle of radius of 200 m. Suppose that channel gains follow a log-distance path loss model with a path loss exponent 2.5. For all SUs, the discounting factor γ is 0.8. In the ϵ-greedy algorithm, ϵ is initially set at 0.8, converging to 0 with the increase of the number of iterations. In this section, each SU uses a feedforward neural network (FNN) that includes three hidden layers and two neurons. The input layer of the FNN has three nodes, including some information such as states and actions taken by neurons, while the output layer has only one node. The capacity of the experience replay memory and the update step size are set to 200 and 10, respectively.

**Figure 1:**
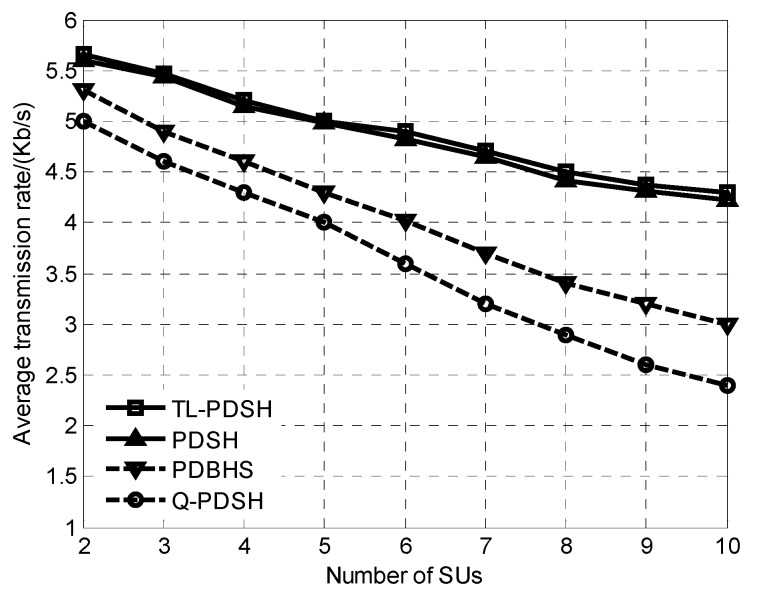
Average transmission rate versus number of SUs.

Figure 1 shows that the average transmission rate $\frac{1}{L}\sum_{i=1}^{L}R_{i}$ versus the number of SUs for *ρ* = 0.9. It can be seen from the simulation results in Figure 1 that the average transmission rate decreases as the number of SU increases. The reason lies in that as the number of SU increases, the interference among SUs will increase, and in order to meet the constraints on SU’s interference with PU and the level of success rate of spectrum handoff, the transmit power of each SU will decrease, and its SINR will also decrease accordingly, resulting in the decrease of each channel capacity. In addition, the simulation results show that the TL-PDSH algorithm can achieve the highest transmission rate while the Q-PDSH algorithm obtains the lowest.

**Figure 2:**
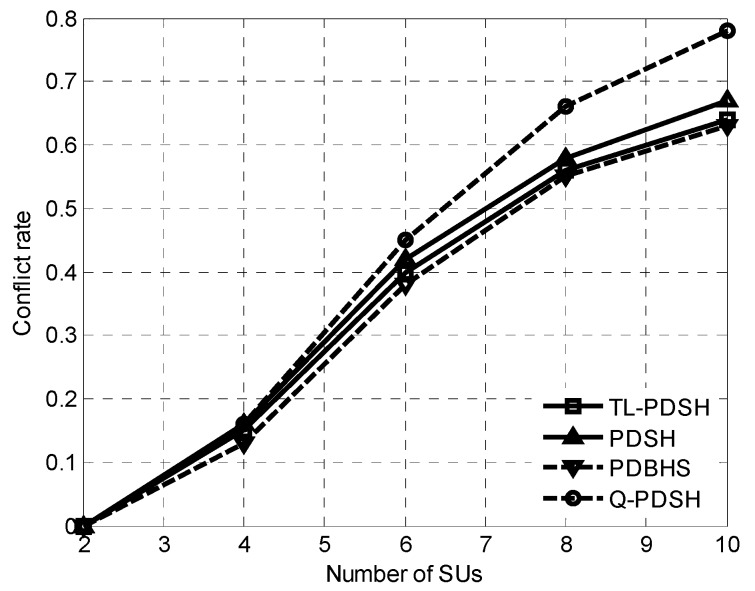
Conflict rate versus number of SUs.

Conflict rate is defined as the percentage of the number of SUs that violate the SINR constraints once or twice while all SUs remain within acceptable level of rewards. Curves of the conflict rate versus the number of SUs for *ρ* = 0.9 is illustrated in Figure 2. It can be seen from the curves that the conflict rate of the four algorithms increases rapidly with the increase of the number of SUs. The number of SUs increases and all SUs pursue the maximum transmission rate, which inevitably results in the increase of the number of SUs violating the SINR constraints, so the conflict rate increases accordingly. In PDBHS method, it selects the channel with the highest probability of getting *K* consecutive idle time-slots as the target channel for spectrum handoff, so the conflict rate of PDBHS is the lowest among the four algorithms. In addition, Figure 2 also shows that the TL-PDSH method is very close to the PDBHS method and the number of SUs needed to achieve the optimal transmit rate can be determined by fixing the conflict rate.

**Figure 3:**
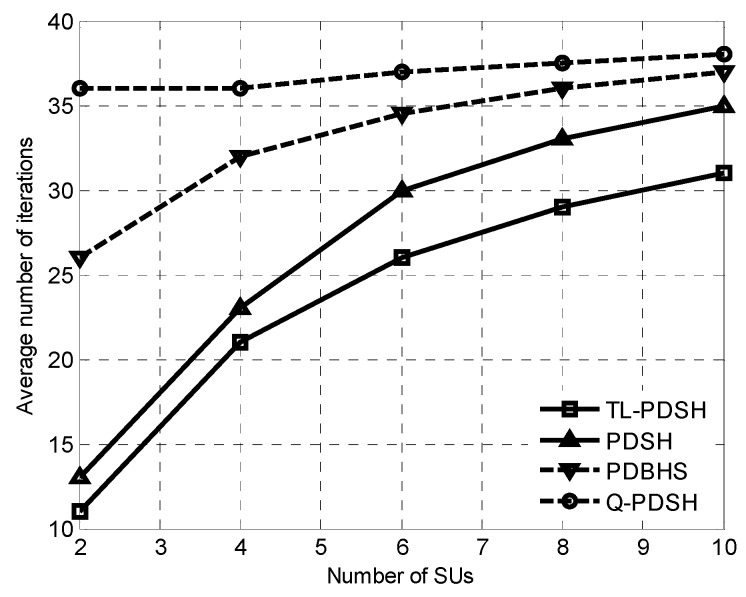
Average number of iterations versus number of SUs.

When the four spectrum handoff algorithms converge, the curve of the average number of iterations with the number of SUs for *ρ* = 0.9 is shown in Figure 3. It can be seen that the number of iterations needed to converge for TL-PDSH and PDSH spectrum handoff schemes using DQN strategy is greatly reduced compared to PDBHS and Q-PDSH. It can also be seen that TL-PDSH algorithm utilizing both DQN and TL has the fastest convergence rate, which matches the theoretical analyses described above since TL can efficiently improve learning speed of the system and reduce the number of iterations by transforming the experienced results of the surrounding SUs to the new SUs.

**Figure 4:**
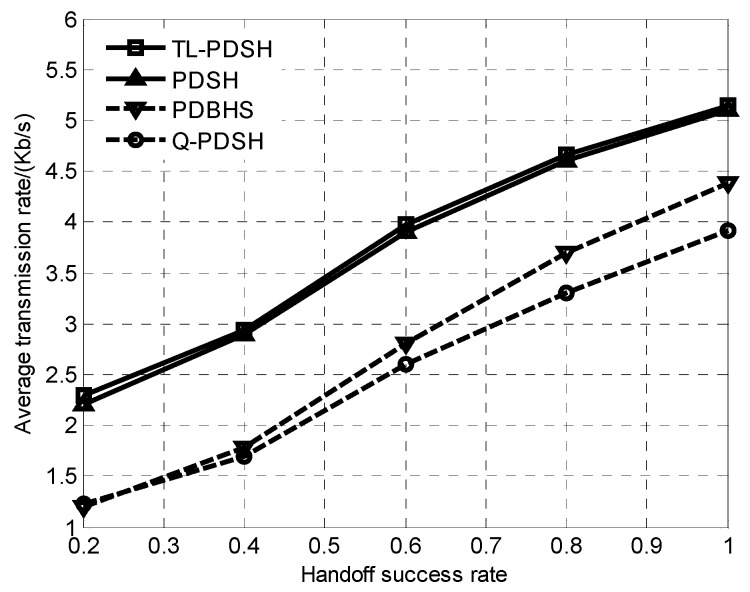
Transmission rate versus handoff success rate.

The average transmission rate of the four algorithms versus handoff success rate for *L* = 6 is demonstrated in Figure 4. The average transmission rate of the four algorithms increases rapidly with the increase of the handoff success rate, which lies in the fact that the higher the probability of spectrum being vacant is, the higher the handoff success rate is, the higher SU’s SINR is, and the higher the channel capacity is. In addition, there is no significant difference between the TL-PDSH algorithm and the PDSH algorithm in terms of average transmission rate performance since both algorithms use the DQN neural learning network.

## 5. Conclusions

In this paper, a DQN neural learning network is used to investigate the spectrum handoff method based on predictive decision in HCRNs, and the TL-PDSH spectrum handoff method based on the DQN predictive decision while meeting the constraints on SUs’ interference with PU and the level of SINR thresholds, as well as the requirements for spectrum handoff success rate is proposed by introducing the spectrum handoff success rate and the TL strategy. Simulation results show that, compared to the existing spectrum handoff methods based on predictive decision, the TL-PDSH method proposed in this paper yields better performances in terms of the system throughput, the handoff success rate and the number of iterations. Furthermore, numeric simulations also demonstrate that under the condition that all SUs seek their own maximum rewards, the conflict rate of our approach is almost the same as that of the PDBHS method which has the minimum conflict rate.
